# Improved Methodology for Assessment of mRNA Levels in Blood of Patients with FMR1 Related Disorders

**DOI:** 10.1186/1472-6890-9-5

**Published:** 2009-06-09

**Authors:** David E Godler, Danuta Z Loesch, Richard Huggins, Lavinia Gordon, Howard R Slater, Freya Gehling, Trent Burgess, KH Andy Choo

**Affiliations:** 1 Chromosome and Chromatin Research, Murdoch Childrens Research Institute, Royal Children's Hospital, Melbourne, Australia; 2 School of Psychological Science, La Trobe University, Melbourne, Australia; 3 Department of Mathematics and Statistics, University of Melbourne, Melbourne, Australia; 4 The Bioinformatics Unit, Murdoch Childrens Research Institute, Royal Children's Hospital, Melbourne, Australia; 5 VCGS Cytogenetics Laboratory, Murdoch Research Institute, Royal Children's Hospital, Melbourne, Australia; 6 Department of Pediatrics, University of Melbourne, Melbourne, Australia

## Abstract

**Background:**

Elevated levels of FMR1 mRNA in blood have been implicated in RNA toxicity associated with a number of clinical conditions. Due to the extensive inter-sample variation in the time lapse between the blood collection and RNA extraction in clinical practice, the resulting variation in mRNA quality significantly confounds mRNA analysis by real-time PCR.

**Methods:**

Here, we developed an improved method to normalize for mRNA degradation in a sample set with large variation in rRNA quality, without sample omission. Initially, RNA samples were artificially degraded, and analyzed using capillary electrophoresis and real-time PCR standard curve method, with the aim of defining the best predictors of total RNA and mRNA degradation.

**Results:**

We found that: (i) the 28S:18S ratio and RNA quality indicator (RQI) were good predictors of severe total RNA degradation, however, the greatest changes in the quantity of different mRNAs (*FMR1, DNMT1, GUS, B2M *and *GAPDH*) occurred during the early to moderate stages of degradation; (ii) chromatographic features for the *18S, 28S *and the inter-peak region were the most reliable predictors of total RNA degradation, however their use for target gene normalization was inferior to internal control genes, of which *GUS *was the most appropriate. Using *GUS *for normalization, we examined in the whole blood the relationship between the *FMR1 *mRNA and CGG expansion in a non-coding portion of this gene, in a sample set (n = 30) with the large variation in rRNA quality. By combining *FMR1 *3' and 5' mRNA analyses the confounding impact of mRNA degradation on the correlation between *FMR1 *expression and CGG size was minimized, and the biological significance increased from p = 0.046 for the 5' *FMR1 *assay, to p = 0.018 for the combined *FMR1 *3' and 5' mRNA analysis.

**Conclusion:**

Our observations demonstrate that, through the use of an appropriate internal control and the direct analysis of multiple sites of target mRNA, samples that do not conform to the conventional rRNA criteria can still be utilized to obtain biologically/clinically relevant data. Although, this strategy clearly has application for improved assessment of *FMR1 *mRNA toxicity in blood, it may also have more general implications for gene expression studies in fresh and archival tissues.

## Background

The mutations in an X linked *FMR1 *gene [1] have some unique qualities. While large CGG expansions (>200) 3' of its promoter give rise to fragile X syndrome – a neurodevelopmental disorder caused by silencing of the gene, smaller expansions (permutations – PM, 55–200 repeats) lead to late onset pathologies through entirely different mechanisms, involving a toxic gain of function of this gene's transcripts [2-4]. Fragile X Tremour Ataxia Syndrome (FXTAS) is the most prevalent disorder associated with PM alleles occurring in 50% of all older carrier males [5]. It is progressive neurodegenerative disorder manifesting with ataxia and/or intension or other tremors, cognitive decline, psychiatric involvement and characteristic MRI and histopathological features [5-8]. In females, these alleles are associated with a significant increase in premature ovarian failure [9]. A toxicity of an elevated *FMR1 *mRNA which is believed to be pathogenic in these conditions, has been supported in animal models where it leads to increased cell death in *Drosophila *[10] and mice [11]. Although, in humans the level of mRNA is usually assessed in whole blood, there is a significant correlation between FXTAS associated with these levels and the typical neurological [12], and psychiatric [13] manifestations. This implies that the assessments of *FMR1 *mRNA levels in carriers of small CGG expansions, apart from being essential in the mechanisms involved in RNA toxicity, may also have a diagnostic and prognostic significance.

*FMR1 *expression is primarily regulated by the state of methylation of its promoter. *DNMT1 *is an enzyme pivotal in this process [14-16]. This implies that DNMT1 may be indirectly involved in the mechanisms leading to *FMR1 *related disorders. Thus, expression of both genes, *FMR1 *and *DNMT1*, is of interest in fresh and archival samples from individuals with diseases characterized by abnormal expansions within the CGG region. Unfortunately, archival samples and samples extracted from blood have variable total RNA and mRNA degradation, which we have found to be a confounding factor in the gene expression analysis, unless a method was established to normalize for *FMR1 *and *DNMT1 *mRNA degradation.

Previous real-time PCR studies normalized gene expression against total cell number, total RNA or mRNA of internal control gene/s, reviewed in [17]. The main constraints associated with using total cell number for normalization is that the RNA has to be of a very high quality and the cell counts have to be accurate, which is not possible for solid tissues. Total RNA has been particularly useful for normalization using absolute and relative standard curve methods for real-time PCR, and has been shown to produce biologically relevant and highly reproducible data [18]. However, the most frequently applied normalization method involves the use of internal control gene/s based on the rational that the mRNA levels of these control genes in the tested samples should reflect the effect of variables on the quantity and quality of the target gene mRNA, and at the same time be minimally effected by the tested variables [19,20]. Previous real-time PCR expression assays for *FMR1 *and *DNMT1 *have utilized *GUS *and *HPRT *as internal control genes [2,21,22]. However, these assays did not take into consideration the appropriateness of these genes as controls in relation to the quantity and quality of the starting material. A number of studies have suggested that confirmation of total RNA quality prior to real-time PCR quantification via capillary electrophoresis is essential in obtaining meaningful gene expression data [23].

In this study the Experion system (Bio-rad) was used to assess the quality of rRNA by subjective/visual interpretation of the chromatographs for absence of peaks other than those of *18S, 28S *and *5S*, and/or by examining the 28S:18S ratio and the RNA Qiality Indicator (RQI) values. However, these approaches have their limitations which we addressed in the study. We also evaluated in depth the use of internal control genes and the capillary electrophoresis features for the normalization of mRNA degradation in partially degraded and intact RNA from patient lymphoblasts and blood. We then defined the best predictor of target gene mRNA degradation, utilizing it for normalization in the whole blood RNA with a high variability of rRNA quality. In this sample set we examined the relationship between the *FMR1 *mRNA and the CGG expansion in a non-coding portion of this gene, and found that by incorporating the 3' and 5' mRNA analysis data from patients with small to intermediate CGG expansions, we have significantly improved upon the current approach used to examine the *FMR1 *mRNA toxicity, demonstrating the clinical relevance of our strategy.

## Methods

### Cell Culture

Fourteen EBV transformed lymphoblast cell lines from healthy controls (*FMR1 *alleles – 6 to 40 repeats); grey zone carriers (41 to 55 repeats), premutation carriers (55 to 200 repeats) and full-mutation carriers (>200 repeats) [24-26] were obtained from the MCRI tissue culture storage repository, or purchased from Coriell (Table 1). The cell-viability counts and cell composition were determined by trypan blue exclusion, using a hemocytometer. Cells were plated at a density of 10^6 ^cell/ml in RPMI medium (Sigma StLuis, MO, USA)/10% Foetal bovine serum (CSL) at 37°C, 5% CO_2 _overnight. Cell pellets were resuspended in RNA lysis buffer, (10 μl β-mercaptoethanol/1 ml of RLT buffer from Qiagen, Hilden, Germany), and stored at -80°C until RNA extraction.

**Table 1:** Cell Line Details

**RNA ID:**	**MCRI Cell Line ID:**	**Source:**	**CGG repeat number**	**Classification**
489	CL880207	MCRI TC.	30	Healthy control male

491	CL0050251	Corriell; GM07174A	30	Healthy control male

492	CL860012	MCRI TC.	30	Healthy control male

488	CL860021	MCRI TC.	30	Healthy control male

494	CL920415	MCRI TC.	31	Healthy control male

584	CL0070217	MCRI TC.	47	Grey zone male

585	CL0070183	MCRI TC.	41	Grey zone male

493	CL0050250	Corriell; GM07174A	103	Premutation male

497	CL0060293	MCRI TC.	170	Premutation male

496	CL970149	MCRI TC.	50; 100	Premutation female carrier

487	CL0050246	Corriell; GM07537A	29; 370	FRAX female carrier

498	CL850085	Corriell; GM4025A	490	FRAX male

586	CL850084	Corriell; GM3200A	530	FRAX male

495	CL840159	MCRI TC.	563; 47	FRAX female

*MCRI TC – Murdoch Childrens Research Institute Tissue Culture Repository.

### Degradation of total RNA

Total RNA was extracted and purified using the RNeasy extraction kit (Qiagen Inc., Hilden, Germany). RNA concentrations were measured in triplicate using a NanoDrop ND-1000 Spectrophotometer, with purity being determined by the A260/A280 ratio using the expected values between 1.8 and 2. Each RNA sample was then diluted to 2 ng/ul. Sixty ul of each sample was left at room temperature for 0, 18, 24 and 96 hours. At each time point an aliquot of 15 ul was collected from each sample, 5 ul of which were used for capillary electrophoresis (Bio-rad) and 2 ul for cDNA synthesis.

### RNA samples from blood of CGG expansion carriers

The original study that investigated the relationship between *FMR1 *expression and CGG expansion was approved by the La Trobe University Human Ethics Committee, the Southern Tasmanian Health and Medical Human Research Ethics Committees, and by the Institutional Review Board of the University of California at Davis. The methods used for CGG repeat sizing and *FMR1 *mRNA assessments, as well as the results of correlation between these two measures in a larger sample, which included the present participants, were reported in the earlier study [4]. An aliquot of total RNA was originally isolated from 3 ml of peripheral blood using Tempus Blood RNA tubes as previously described in: Loesch, et al. (2007) [4]. After more than 1 year of storage at -80°C, the total RNA quality was assessed using capillary electrophoresis. All samples were diluted to 5 ng/ul, prior to reverse transcription. Here we utilized these earlier data on CGG repeat size, but validated and conducted new assays on the expression of *FMR1ex3.4, FMR1ex13.14 *and *GUS*.

### Capillary electrophoresis

**Figure 1:**
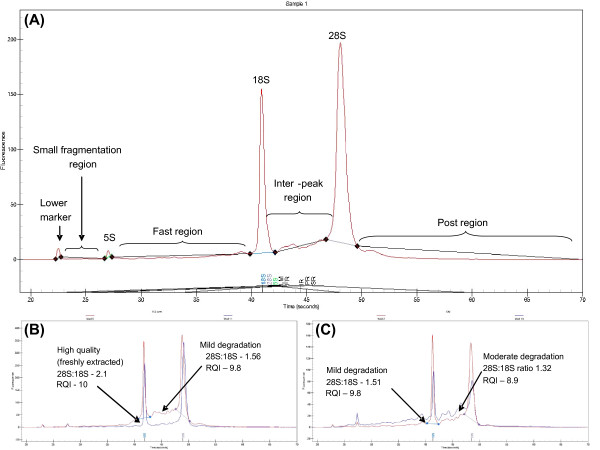
**Assessment of chromatograms for total RNA degradation**. (A) *Objective assessment *– (manually set parameters) included percentage area under the lower marker (LM), small fragment region (SR), 5S, fast region (FR), 18S, the inter-peak region (IR), 28S, post region (PR) and the 28S/18S ratio. (◆) defines the boundary of each of the regions. (B) *Subjective assessment *of the same sample before (blue) and after (red) being degraded at room temperature. The typical features included increase in baseline (primarily IR), decrease in 28S% area, 28S:18S ratio and RQI. (C) *Subjective assessment *of the same sample moderately (red) and highly (blue) degraded at room temperature. The typical features included increase in the baseline (FR, IR, SR), increase in 5S, and decrease in 18S% and 28S% areas as well as the 28S:18S ratio and RQI.

Total RNA quality was assessed using the RNA HighSens Kit as per manufacturer's instructions (Bio-rad). Objective and subjective analysis of each RNA profile was performed. Objective assessment involved automatic collection of a number of (manually set) parameters which included percentage area under the lower marker (LM), small fragment region (SR), 5S, fast region (FR), 18S, Inter region (IR), 28S, post region (PR), the 28S:18S ratio and RQI (Figure 1A). Subjective assessment (Figure 1B and 1C) involved descriptive comparison of chromatographic features based on previous publications using this system [27].

### Reverse Transcription Real-time PCR

Reverse transcription was performed one reaction per sample using the Multiscribe Reverse Transcription System, 50 units/ul (Applied Biosystems). The 7900HT Fast Real Time PCR (Applied Biosystems) was used to quantify *FMR1ex3.4, FMR1ex13.14, DNMT1, GAPDH, B2M*, and *GUS*, using the relative standard curve method. The target gene and the internal control gene dynamic linear ranges were performed on a series of doubling dilutions of RNA (160-0.5 ng/ul) of a selected peripheral bood mononuclear cell sample. Previously published sequences were used for RT-PCR primers and TaqMan probe for: *FMR1exon3/4 *and *GUS *[2]; *FMR1exon13/exon14 *[21], and *DNMT1 *[22]. *FMR1exon3/4, FMR1exon13/14 *and *DNMT1 *primers and probes were used at concentrations of 18 uM and 2 uM, respectively. *GAPDH *and *B2M *primer/probe mixes were obtained from PrimerDesign (PerfectProbe ge-PP-12-hu kit) and used at concentration of 2 uM. Each sample was assayed in duplicate 10 μl PCR reactions, consisting of 5.8 mM MgCl_2_, 1 μl Buffer A (Applied Biosystems), 3.35 μl RNase-free water, 1.2 mM dNTPs, 0.01 units/μl of AmpliTaq Gold, 0.5 μl of TaqMan probe and 0.5 μl forward and 0.5 μl reverse primers, and 1 μl of the reverse transcription (cDNA) reaction. The annealing temperature for thermal cycling protocol was 60°C for 40 cycles. Samples were quantified in arbitrary units (au) in relation to the standard curves performed on each plate.

### Statistical analysis

Generalized estimating equations (GEE) were used for the objective assessment of the chromatographs and mRNA integrity. Normalization of *FMR1 *and *DNMT1 *mRNA to *18S, 28S, GAPDH *and *GUS*, expressed as a function of the degradation time, was assessed by fitting the values to a simple linear regression. The sign test was then used to examine the null hypothesis that the median slope was zero against the two-sided alternative of it not being equal to 0. The relationship between the qPCR results for *FMR1ex3.4 *and *FMR1ex13.14 *assays with the CGG expansion size was assessed using a significance test in a linear regression. The analysis was conducted using the publicly available R statistical computing package, version 1.191 (R development Core team, 2004).

## Results

### Subjective assessment of chromatographs and mRNA integrity of FMR1 and DNMT1

**Figure 2:**
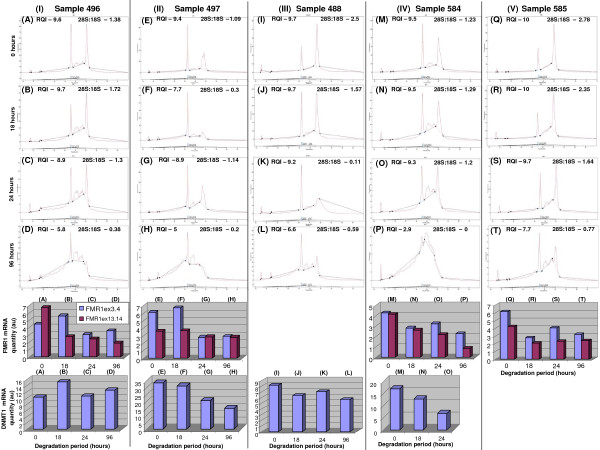
**Relationship between subjective assessment of chromatograms for total RNA degradation and quantification of *FMR1 *and *DNMT1 *mRNA, using real-time PCR**. RNA samples from 5 lymphoblast cell lines were degraded at room temperature for 0 hr (A, E, I, M, Q), 18 hrs (B, F, J, N, R), 24 hrs (C, G, K, O, S), and 96 hrs (D, H, L, P, T), and assessed for total RNA integrity using Experion capillary electrophoresis ststem. Panels I-IV – RNA samples 496, 497, 488, 584, and 585, respectively. (◆) defines the boundary of each of the regions (see Figure 1). *FMR1ex3/4*, *FMR1ex13/14 *and *DNMT1 *mRNA arbitrary quantities were determined using real-time PCR relative standard curve method. Samples with the coefficient of variance greater than 30% between the duplicate reactions were omitted from the analysis.

*FMR1 *and *DNMT1 *mRNA quantities were examined using the relative standard curve method, in RNA samples 496, 497, 488, 584 and 585 artificially degraded at room temperature for 0, 18, 24 and 96 hours from 5 lymphoblast cell lines (Table 1). The samples were subjectively assessed as described in Figure 1 for the relationship between the capillary electrophoresis profiles and mRNA quantities of *FMR1 *(5' and 3') and *DNMT1 *(Figure 2).

Sample 496 (Figure 2; Panel I) displayed minor differences in the capillary electrophoresis profiles between 0 and 24 hours, with the most prominent change being observed between 24 and 96 hours (a large increase in the inter-peak area and the fast region, and a marked decrease in the *28S *peak). This was also reflected in the 28S:18S ratios and RQI values, where there was no change between 0 and 24 hours, but a large decrease between 24 and 96 hours. In contrast, *FMR1ex13.14 *mRNA level decreased by more than half within the first 18 hours and remained at this level for the following time points. A similar trend was observed for *FMR1ex3.4 *mRNA, which decreased between 18 and 24 hours of incubation, and remained at the same level through to 96 hours. In contrast, there were no major changes in *DNMT1 *mRNA quantities for sample 496 throughout the time course.

Sample 497 was different to all the other RNA examined, as it displayed a second, smaller *28S *peak at 0 hours (Figure 2; Pannel II E). The origin of this peak, which has been previously reported using the analogous system (Schroeder et al.; United states patent publication 2006/0246577 A1) is unknown; the absence of any other RNA degradation markers such as an increase in the inter-peak area and/or the fast region argue against a plausible explanation that it could represent 28S rRNA degradation. It is also unclear why a significant change in the capillary elecrophoresis profile for this sample was observed at 18 hours, when the larger 28S peak(s) almost completely disappeared (Figure 1, Panel II F), while at 24 hours there was an increase in the 28S:18S ratio and RQI (Figure 1, Panel II F and G). This may be linked to the structure of the anomolous 28S peak, the unusual integrity of which is beyond the scope of this study. Interestingly, the mRNA levels for *FMR1 *and *DNMT1 *did not change between 0 and 18 hours. The greatest increase in total RNA degradation of sample 497 was observed between 24 and 96 hours, as indicated by a significant increase in the inter-peak area and the fast region and a marked decrease in the 28S:18S ratio and the RQI value (Figure 2, Panel II G and H). However, again this trend was not mirrored by the degradation of *FMR1 *and *DNMT1 *mRNA, which showed no major differences between 24 and 96 hours.

For the sample 488, within the first 18 hours there was no difference in RQI, however the 28S:18S ratio decreased from 2.5 to 1.57, with a slight increase in the inter-peak area within the first 18 hours (Figure 2, Panel III, I and J). At 24 and 96 hours we observed a further decrease in the 28S:18S ratio which was mirrored by a moderate decrease in RQI, and a prominent increase in the inter peak area and the fast region (Figure 2, Panel III, K and L). In contrast, there was only a slight decrease in *DNMT1 *mRNA level within the first 18 hours, which remained at the same level for the following time points.

Samples 584 and 585 demonstrated similar kinetics of total RNA degradation. At 0 hours these samples had similar RQI values, but different 28S:18S ratios (Figure 2, Panels IV and V). A striking increase in total RNA degradation was observed between 24 and 96 hours. During this period for sample 584 the 28S:18S ratio dropped from 1.2 to 0, and RQI from 9.3 to 2.9 (Figure 2, Panels IV; O and P), while for sample 585 the 28S:18S ratio dropped from 1.64 to 0.77, and RQI from 9.7 to 7.7 (Figure 2, Panel V; S and T). This was related to a significant increase in the inter peak area and the fast region. In contrast, mRNA quantities of *FMR1ex3.4 *and *FMR1ex13.14 *were decreased by approximately half between 0 and 18 hours in both samples 584 and 585, and for *DNMT1 *in sample 584 about 3 fold between 0 and 24 hours.

The similarities in FMR1 mRNA and total RNA degradation kinetics in both 584 and 585 samples may be related to these cell lines harbouring grey zone alleles, whereas samples 496 and 497 that showed different degradation kinetics were from premutation carriers (Table 1). Although, an in-depth investigation of this relationship is beyond the scope of this manuscript, the differences in FMR1ex3.4 mRNA degradation kinetics between the premutation and grey zone cell lines (Figure 2) were consistent with previous studies showing that the increased length of the CGG tract correlates with increased mRNA stability through hairpin formation within the 5'UTR repeat region [28].

Together these data suggested that the total RNA degradation rate moderately varies between the samples from different cell lines with the most prominent changes being observed between 24 and 96 hours. This was poorly correlated with the profile of *FMR1 *and *DNMT1 *mRNA degradation that predominantly occurred within the first 18 to 24 hours, indicating that there was no clear correlation between the rate of total RNA degradation from the subjective assessment of the chromatographs and mRNA degradation as determined by real-time PCR. Because FMR1 mRNA stability may be related to the size of the CGG repeat within its UTR and the pathology of FMR1 related disorders [28], in the following sections we have established a method to normalize for mRNA degradation independent of the CGG expansion size, so that the clinical relevance of the CGG related FMR1 mRNA toxicity can be identified in samples with variable rRNA quality.

### Objective assessment of the chromatographs and mRNA integrity

Objective assessment of total RNA and mRNA degradation was performed in RNA samples from 14 cell lines (Table 1). Ten features were measured from each chromatograph and 6 variables measured using real-time PCR from the corresponding cDNA samples at 4 paired time points. The RNA from three fragile X cell lines were excluded from the FMR1 real-time PCR analysis for the RNA degradation study, as they had no FMR1 expression. The first aim of this approach was to objectively delineate which features of the chromatographs and gene expression profiles (*GAPDH, B2M, GUS*) could be used as predictors of the total RNA degradation as reflected by the degradation time. The second aim was to objectively delineate whether *FMR1 *and *DNMT1 *mRNA degradation correlated with the degradation time, and if not, which features could be used as predictors of the target gene mRNA integrity.

Based on the subjective assessment, we arbitrarily divided the degree of total RNA degradation into four categories: 0 hours – intact; 18 hours – early degradation; 24 hours – moderate to severe degradation; 96 hours – severe degradation, under the assumption that RNAs from different cell lines follow this progressive trend of degradation at the four time points. GEE were then utilised to provide an estimation of which parameters most closely reflected *FMR1 *and *DNMT1 *mRNA integrity in early versus moderate versus late degradation stages, and through the time course as a whole.

As expected, the most significant predictors of total RNA degradation from the combined and individual comparisons of chromatographic features were the percentage areas of 18S, 28S and the inter-peak region (Table 2). These features appeared to be suitable predictors of early, moderate and severe RNA degradation (p < 0.001). In contrast, 5S % area was a good predictor of only early to moderate RNA degradation (p < 0.05), and the small fragmentation region percentage area of moderate to severe RNA degradation (p < 0.05). However, the most prominent predictors of severe RNA degradation were the fast region % area (p < 0.001), the 28S:18S ratio (p < 0.001), the RQI (p < 0.001) and the lower marker % area (p = 0.072). Interestingly, in contrast to the 28S:18S ratio and the lower marker % area, the RQI could be also used as a predictor of moderate degradation (p < 0.05). The post region % area was the only parameter that was not associated with any stage of RNA degradation.

**Table 2:** Correlation of RNA degradation time with capillary electrophoresis features and mRNA quantities

***RNA feature***	***Predominant size of*** ***RNA detected and*** ***amplicon length*** ***(for mRNA only)***	***0 to 18 hours comparison*** ***p values***	***0 to 24 hours comparison*** ***p values***	***0 to 96 hours comparison*** ***p values***	***Combined comparison*** ***p values***
18S % of total area (n = 53)	1700 bp	0.001***	0.001***	0.001***	0.001***

28S % of total area (n = 53)	3770 bp	0.001***	0.001***	0.001***	0.001***

5S % of total area (n = 53)	160 bp	0.043**	0.001***	0.386	0.001***

Fast region % of total area (n = 53)	____	0.717	0.92	0.001***	0.001***

Inter-peak region % of total area (n = 53)	____	0.001***	0.001***	0.001***	0.001***

Post region % of total area (n = 53)	____	0.36	0.16	0.316	0.42

Lower Marker % of total area (n = 53)	____	0.256	0.322	0.072*	0.001***

Small fragmentation region % of total area (n = 53)	____	0.12	0.046**	0.074*	0.026**

28S:18S ratio (n = 53)	____	0.553	0.082*	0.001***	0.001***

RQI	____	0.223	0.024**	0.001***	0.001***

*DNMT1 *mRNA (au) (n = 41)	5084 bp – 142 bp	0.173	0.006**	0.004**	0.001***

FMR1ex.3.4 mRNA (au) (n = 43)	4350 bp – 122 bp	0.265	0.001***	0.001***	0.003**

*FMR1ex13.14 *mRNA (au) (n = 43)	4350 bp – 89 bp	0.024**	0.013**	0.012**	0.001***

*GUS *mRNA (au) (n = 52)	2200 bp – 80 bp	0.169	0.001***	0.001***	0.001***

*B2M *mRNA (au) (n = 35)	976 bp – 114 bpo	0.093*	0.13	0.571	0.01**

*GAPDH *mRNA (au) (n = 52)	1310 bp – 110 bp	0.117	0.001***	0.001***	0.001***

*Generalized estimating equations (GEE) were used. Data from samples with different levels of total RNA degradation (early, moderate, high) are shown as the p-values of the chi square test (p < 0.1*; p < 0.05**; p < 0.001***) and the sample size (n).

*GUS *and *GAPDH *mRNA degradation closely reflected moderate to severe degradation of total RNA (p < 0.001), best represented by the small fragmentation region % area. In contrast, *B2M *mRNA could only be used to predict early total RNA degradation (p = 0.093). Furthermore, it was not associated with any other chromatographic and gene expression parameters examined. For the target transcripts, *DNMT1 *and *FMR1ex3/4*, mRNA degradation was closely associated with moderate to severe degradation of total RNA (p < 0.001), and was best predicted by the small fragmentation region % area, and by *GUS *and *GAPDH *mRNA, all of which had similar degradation kinetics. In contrast, *FMR1ex13.14 *mRNA degradation appeared to be closely associated with early, moderate and severe total RNA degradation (p < 0.001), and was best predicted by % areas of 18S, 28S and the inter-peak region (p < 0.001). This analysis demonstrated that the degradation of mRNA was different between most internal controls and target genes examined, and that the degradation kinetics of specific mRNAs were not necessarily the same as those for total RNA and rRNA.

### Assessment of FMR1 and DNMT1 mRNA normalized to capillary electrophoresis and real-time PCR predictors of mRNA degradation

**Figure 3:**
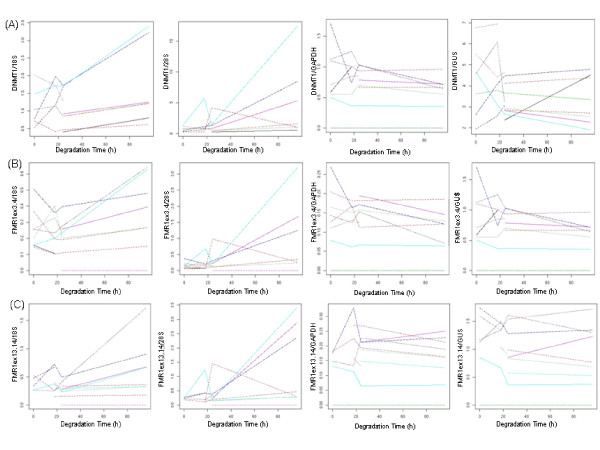
**Normalization of *FMR1 *and *DNMT1 *mRNA to 18S, 28S, *GAPDH *and *GUS*, expressed as a function of degradation time**. Each sample (listed in Table 1) is represented by a differently colored line whose number varied between 8 and 14 for the nine plots; depending on completeness of the data set for the variables examined. (A) *DNMT1 *mRNA normalized to 18S (p = 0.066), 28S (p = 0.066), *GAPDH *(p = 0.388), *GUS *(p = 0.774); (B) *FMR1ex3.4 *mRNA normalized 18S (p = 0.11), 28S (p = 0.11), *GAPDH *(p = 0.51), *GUS *(p = 0.254); (C) FMR1ex13.14 mRNA normalized 18S (p = 0.11), 28S (p = 0.11), *GAPDH *(p = 0.11), *GUS *(p = 0.34).

The target gene expression in the RNA samples from the cell lines were normalized to the best predictors of *FMR1 *and *DNMT1 *mRNA degradation as determined by the objective assessment of chromatographs and mRNA integrity. 18S % area, 28S % area, *GUS *mRNA or *GAPDH *mRNA quantity were used for normalization, and expressed as a function of the degradation time. The normalization method that provided the most constant (least significant) values throughout the time course was considered as the most optimal of the predictors tested for the target gene mRNA degradation (Figure 3; the sign test was used to examine the null hypothesis that the median slope was zero against the two-sided alternative of it not being equal to 0). We found that for *DNMT1 *both 18S (p = 0.066) and 28S (p = 0.066) % area were poor normalization features compared to *GAPDH *(p = 0.388) and *GUS *(p = 0.774). Similarly, for *FMR1ex3.4*, both 18S (p = 0.11) and 28S (p = 0.11) % area normalization provided less constant values than *GAPDH *(p = 0.51) and *GUS *(p = 0.254). For *FMR1ex13.14*, 18S (p = 0.11) and 28S (p = 0.11) % area as well as *GAPDH *(p = 0.11) normalization provided less constant values than *GUS *(p = 0.34). Thus, it appeared that normalization of the target genes to 18S and 28S chromatographic features was overall inferior to the use of internal control genes, *GAPDH *and *GUS*. Although 18S and 28S % areas could be still be used as normalization markers for *FMR1ex3.4 *and *FMR1ex13.14*, the most optimal of the predictors tested for both *DNMT1 *and *FMR1 *was *GUS*.

### Combining the analysis of 3' and 5' mRNA sites and GUS normalization minimizes the confounding impact of mRNA degradation in RNA samples from whole blood with highly variable rRNA quality

We have previously demonstrated using freshly extracted RNA, that *FMR1 *expression was significantly elevated in carriers of CGG expansion, compared with normal controls of a similar age, and that the expression was proportional to the size of CGG expansions within the grey zone and lower premutation range [4]. Subsequent analysis of these stored samples revealed a high variability in rRNA quality, which posed major confounder concerns. We have therefore determined whether these samples could provide clinically relevant data using the normalization criteria tested in the study.

**Figure 4:**
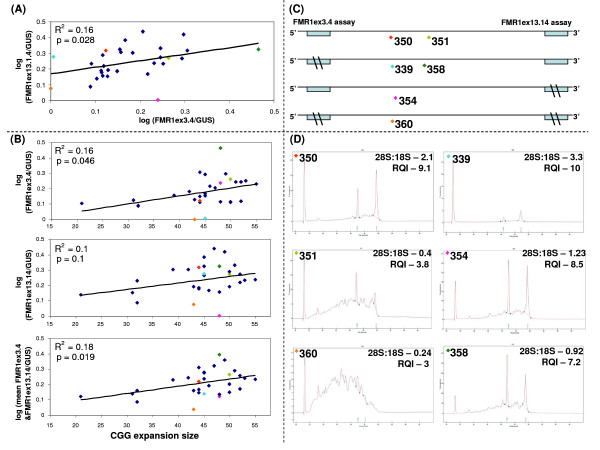
**Relationship between *FMR1 *mRNA degradation, rRNA integrity and biological relevance of expression data in RNA samples from patient whole blood (n = 30) with CGG expansion between 20 and 55 CGG repeats**. (A) Relationship between qPCR results for *FMR1ex3.4 *(x axis) and *FMR1ex13.14 *assays (y axis) standardized to *GUS*. (B) Relationship between CGG expansions (x axis) and qPCR results standardized to *GUS *(y axis) for *FMR1ex3.4 *and *FMR1ex13.14 *assays separately and combined. (C) Representation of proposed locations for mRNA breaks and loss of molecules for qPCR (\\) within product sequences of both assays for color coded representative samples (350 and 351) and outliers (339, 360, 354 and 358). (D) Chromatographs, 28S:18S and RQI values for color coded samples (each dot of the same color represents the same sample).

The *FMR1ex3.4*/*GUS *mRNA levels assessed here closely corresponded to the levels of the earlier study in freshly extracted RNA samples[4], but using the relative standard curve as opposed to the delta-Ct method. As expected we have also found a significant correlation (p = 0.028) between the *FMR1ex3.4/GUS *and *FMR1ex13.14/GUS *assays indicating that most samples had intact *FMR1 *mRNA (Figure 4A) despite the observed variability in rRNA quality as exemplified by the chromatographs of samples 350 and 351, with the 28S:18S ratio between 2.1 and 0.4, and RQI between 9.1 and 3.8 (Figure 4C and 4D). We have also found a significant linear correlation between *FMR1ex3.4/GUS *mRNA levels and the CGG expansion size, (p = 0.046) (Figure 4B) indicating the biological relevance of the data. However, there was no significant correlation between *FMR1ex13.14/GUS *mRNA levels and the CGG expansion size (p = 0.1) (Figure 4B).

A number of samples (samples 339, 354, 358 and 360 – colour coded), did not follow the common pattern of correlation between the *FMR1ex3.4/GUS *and *FMR1ex13.14/GUS *assays (Figure 4A), and the *FMR1ex3.4/GUS *and *FMR1ex13.14/GUS *assays with the CGG expansion size (Figure 4B). Two of these samples, 339 and 360 were also outliers for rRNA quality assessment with the 28S:18S ratios of 3.3 and 0.24, and RQI values of 10 and 3, respectively (Figure 4D). Furthermore, there was no uniform correlation between rRNA integrity and *FMR1 *mRNA quality for both the *FMR1ex3.4/GUS *and *FMR1ex13.14/GUS *assays in these samples. Each of these samples appeared to be significantly affected at either the 5' or 3' sites (Figure 4A, B and 4C). Only one sample (360), with poor rRNA profile, appeared to have *FMR1 *mRNA integrity compromised at both the 5' and 3' sites (Figure 4B and 4D). The confounding impact of these outliers was minimized when the data for the *FMR1 *mRNA 3' and 5' end analyses were combined, as the significance of the *FMR1 *correlation with CGG expansion size increased to p = 0.018.

## Discussion

The specific aim of this study was to establish an optimal method to normalize for the degradation of target gene *FMR1 *mRNA in a sample set showing large variability in rRNA quality, and demonstrate it's clinical/biological relevance. Previous studies have normalized *FMR1 *expression to *GUS *[2,21]. However, we questioned the appropriateness of *GUS *as an internal control for our sample set, where we have observed a large variation in rRNA degradation, particularly as there are no previous studies that examined the rates of mRNA degradation for both *FMR1 *and *GUS*. Since different mRNA species degrade depending on their length and secondary structure [18,23,29], we assessed if *GUS *was a suitable control for *FMR1 *mRNA degradation, and if not, which of the capillary electrophoresis and real-time PCR parameters would provide a better normalization method. Because of the potential relevance of *DNMT1 *to *FMR1 *gene regulation [30-33], we have included a parallel analysis of *DNMT1 *as as target gene in some of the studies.

We initially demonstrated using the Experion system that for the artificially degraded RNA samples, both the 28S:18S ratio and the RQI were most useful as predictors of severe RNA degradation, whereas the greatest changes in stability of different transcripts examined occurred during early to moderate stages of RNA degradation. Thus, the 28S:18S ratio and the RQI were not suitable predictors of mRNA stability, at least in our settings. Since, the RQI is closely related to a more widely used RNA Integrity Number (RIN) from an analogous Agilent system (Bio-Rad electrophoresis technical note 5761), our findings suggest that RIN may be also inappropriate as a normalization tool in our settings. Furthermore, the subjective assessment of general chromatographic features did not provide a useful estimation of mRNA degradation, as real-time PCR could still be used to obtain biologically relevant mRNA data in samples with chromatographs indicating severe rRNA degradation. In another approach, we determined that of the 10 selected chromatographic features, 18S, 28S and the inter-peak region % areas were the most reliable predictors of total RNA degradation when examined as a function of the degradation time. However, the normalization of the target genes to 18S and 28S chromatographic features was found to be inferior to the use of the internal control genes.

Primarily these observations indicate that the degradation kinetics of rRNA may be heavily size dependent, as the small 5S rRNA subunit, 160 nucleotides, was found to be a good predictor of only early to moderate RNA degradation, whereas the 18S and 28S % areas, 1770 and 3770 nucleotides respectively, could be used to predict RNA degradation at early, moderate and late stages of degradation. For mRNA quantitation, by qPCR, however, it has been previously suggested that the length of the amplicon, rather than that of the whole mRNA molecule, may be a more important indicator of degradation kinetics, particularly because fragmentation of a long mRNA may only result in a loss of the molecule for qPCR detection if the RNA break occurs within the product sequence [23].

We've found that the location of the amplicon, may be just as important as the size in determining the effect of mRNA degradation on qPCR performance. This was initially observed by examining the differences in *FMR1ex3.4 *(5') and *FMR1ex13.14 *(3') qPCR data throughout the RNA degradation time course. Since most *FMR1 *transcripts contain the 195-bp exon 14 [34], and all contain exons3/4, both assays target *FMR1 *transcripts of a similar size and abundance, which is experimentally reflected by the highly significant correlation between the two assays for high quality total RNA samples from the peripheral blood of patients with small to intermediate size expansions (Additional file 1). Since the amplicons for both assays are also of a similar size, if the location of the amplicon is unimportant in determining RNA degradation kinetics, its effects should be similar on the qPCR results for both assays. However there were clear differences between the 5' and 3' *FMR1 *results for the sample set with the high variability in total RNA quality, which may be explained by the 3' alternative splice site being more susceptible to RNA breaks than the 5' common region. Thus, thermal stress at the 3' site may result in the loss of more molecules for qPCR detection, than that at the 5' site, which is consistent with our findings in the RNA samples extracted from whole blood where RNA degradation had far less impact on the biological significance of the *FMR1ex3.4 *data than on the *FMR1ex13.14 *results (Figure 4B).

We have also found that due to the differences between degradation kinetics of mRNA and rRNA, normalization of target mRNA levels using rRNA profiles, as indicated by capillary electrophoresis, was inferior to the use of the internal control genes. Therefore, we proposed an alternative approach, independent of capillary electrophoresis, where by targeting each transcript at multiple sites for qPCR analysis, the confounding impact of mRNA breaks at any specific location on qPCR performance can be minimized. Specifically, in a previous study we've found that the *FMR1 *mRNA levels were proportional to the size of CGG expansions within the grey zone and lower PM range [4]. In 30 of the original samples we've found the rRNA quality to vary significantly. Half of the samples would have had to be omitted from further analysis unless an alternative method to capillary electrophoresis was found to normalize for mRNA degradation.

We examined *FMR1 *expression in these samples without omission by targeting *FMR1 *transcript at 5' and 3' ends standardized to *GUS*, as we have shown that this strategy is superior to the use of rRNA profiles from the capillary electrophoresis analyses of the artificially degraded samples. Of the 30 samples we found four outliers that had a significant impact on qPCR performance particularly for the 3' *FMR1ex13.14 *assay. In 3 out of the 4 outliers this was not directly related to rRNA quality. By combining *FMR1 *mRNA 3' and 5' end analyses, the confounding impact of these outliers on the correlation between *FMR1 *expression and CGG size was minimized, and the statistical significance of the data doubled.

## Conclusion

In summary, we have demonstrated that in artificially degraded RNA samples a number of chromatographic features including 18S, 28S, the inter-peak region, the 28S:18S ratio and RQI can be used as predictors of different stages of total RNA degradation. However, their use for normalization of target gene mRNA degradation was inferior to the use of internal control genes, of which *GUS *was the most appropriate as it closely reflected the target gene mRNA degradation kinetics. For the target *FMR1 *mRNA we've shown that the location of the amplicon, may be just as important as the size of its transcript in determining the effect of mRNA degradation on qPCR performance. Furthermore, we've found that by targeting the *FMR1 *transcript at multiple sites, the confounding impact of mRNA breaks due to fragmentation within any specific qPCR product sequence, and the subsequent loss of mRNA molecules for qPCR detection, was minimized. In clinical practice, this approach may be extremely useful, as there is extensive variation in the time lapse between the blood collection and RNA extraction for different samples, which contributes variation in rRNA and mRNA quality. Our strategy allows for a more accurate comparison between samples without sample loss.

Whilst we have shown that this approach is applicable for the *FMR1 *mRNA in RNA extracted from whole blood in our patient subset, there are also potential implications on the development of diagnostic tests for the levels of *FMR1 *mRNA toxicity associated with a number of clinical conditions [2-9,12,13]. These findings are also likely to have a broader application to expression studies of other genes using precious archival or fresh blood samples with a large variation in rRNA quality where sample omission is not an option. More importantly, the approach presented in this manuscript may be useful to any diagnostic application where mRNA integrity may be compromised [35], including future developments of methods for detection of placental mRNA in maternal plasma, foetal RNA markers for non-invasive prenatal diagnosis of pregnancy associated diseases and foetal chromosomal aneuploidies, as well as clinical uses of plasma or whole blood RNA for detection of tumour RNA in cancer diagnostics.

## Competing interests

The authors declare that they have no competing interests.

## Authors' contributions

DEG conceived the project and performed the majority of the experimental procedures, carried out drafting and writing the manuscript, and prepared the figures. DZL contributed to acquisition of patients' data and RNA samples and was involved in writing and drafting the manuscript and data interpretation. RH and LG conceived and performed all statistical calculations, significantly contributed to data interpretation and were involved in drafting of the manuscript and figure/table preparation. HRS was involved in acquisition of patients' CGG expansion size data, in writing and drafting the manuscript. FG and TB were involved in some aspects of experimental procedures including RNA extractions and real-time PCR analysis, as well as CGG expansion size analysis. KHAC provided guidance in the study, was involved in writing and drafting the manuscript, data interpretation and figure preparation. All authors were involved in critically revising the manuscript in progress, read and approved the final manuscript.

## Pre-publication history

The pre-publication history for this paper can be accessed here:



## Supplementary Material

Additional file 1 ***FMR1ex3.4 *and *FMR1ex13.14 *assays detect transcripts of a similar abundance in peripheral blood of patients (n = 54) with small to intermediate size expansions in RNA samples of high total RNA quality.** Click here for file
