# Supplementary material for: Improved Methodology for Assessment of mRNA Levels in Blood of Patients with FMR1 Related Disorders
Source: BMC Clin Pathol. 2009 Jun 9;9:5. doi: 10.1186/1472-6890-9-5 (PMC2708186; doi:10.1186/1472-6890-9-5)
Supplement: Additional file 1 — FMR1ex3.4 and FMR1ex13.14 assays detect transcripts of a similar abundance in peripheral blood of patients (n = 54) with small to intermediate size expansions in RNA samples of high total RNA quality. [file 1472-6890-9-5-S1.ppt]

## Slide 1
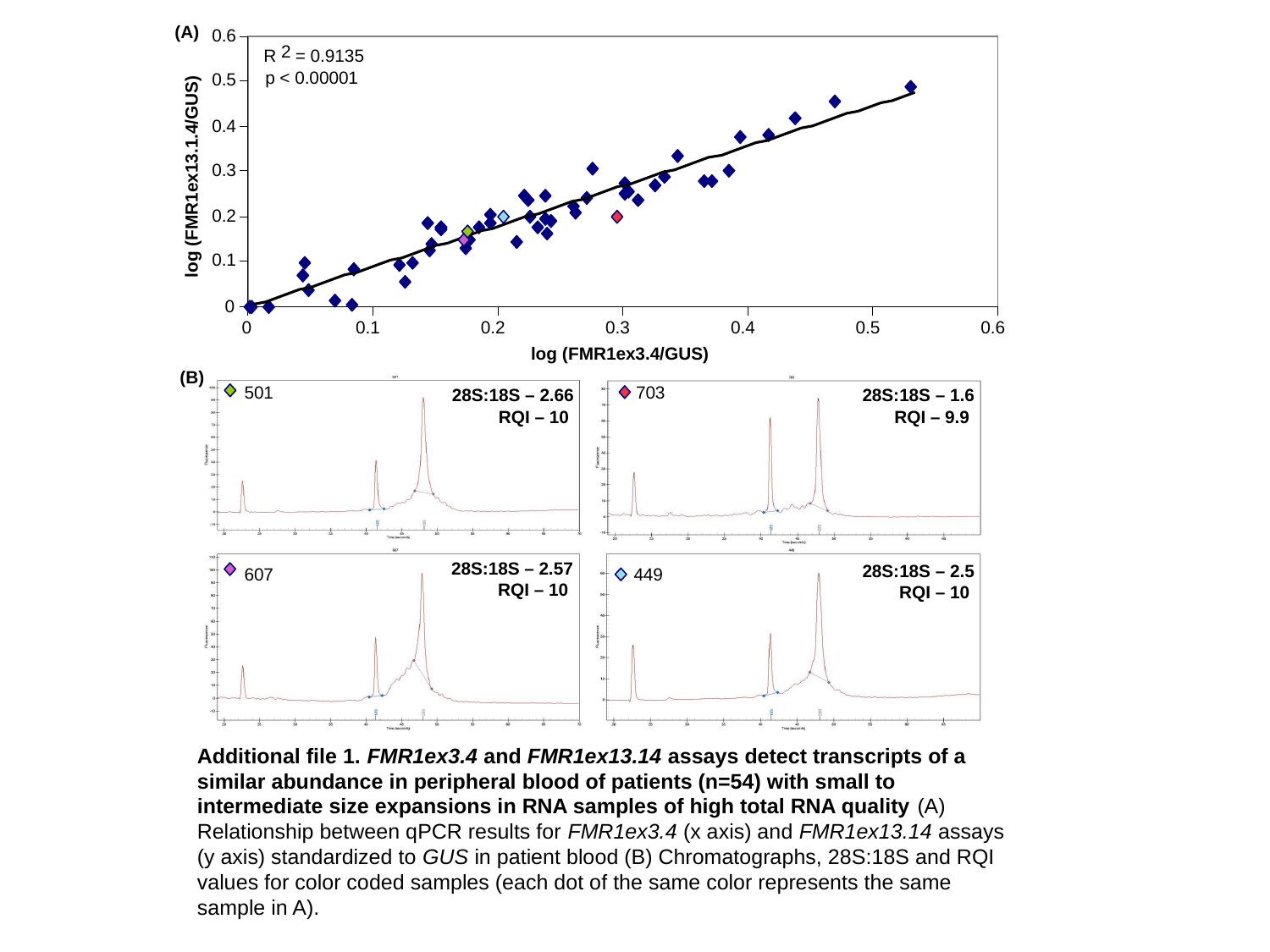

(A)
0.6
2
R
 = 0.9135
p < 0.00001
0.5
0.4
0.3
log (FMR1ex13.1.4/GUS)
0.2
0.1
0
0
0.1
0.2
0.3
0.4
0.5
0.6
log (FMR1ex3.4/GUS)
(B)
501
703
28S:18S – 2.66
 RQI – 10
28S:18S – 1.6
 RQI – 9.9
28S:18S – 2.57
 RQI – 10
28S:18S – 2.5
 RQI – 10
607
449
Additional file 1. FMR1ex3.4 and FMR1ex13.14 assays detect transcripts of a similar abundance in peripheral blood of patients (n=54) with small to intermediate size expansions in RNA samples of high total RNA quality (A) Relationship between qPCR results for FMR1ex3.4 (x axis) and FMR1ex13.14 assays (y axis) standardized to GUS in patient blood (B) Chromatographs, 28S:18S and RQI values for color coded samples (each dot of the same color represents the same sample in A).
